# Junior faculty core curriculum to enhance faculty development

**DOI:** 10.1017/cts.2016.29

**Published:** 2017-02-27

**Authors:** Ronnie Guillet, Robert G. Holloway, Robert A. Gross, Katie Libby, Janine R. Shapiro

**Affiliations:** 1 Department of Pediatrics, University of Rochester, Rochester, NY, USA; 2 Departments of Neurology and Pharmacology & Physiology, University of Rochester, Rochester, NY, USA; 3 Clinical and Translational Science Institute, University of Rochester, Rochester, NY, USA; 4 Department of Anesthesiology, University of Rochester, Rochester, NY, USA

**Keywords:** Mentorship, Career Advancement, Education

## Abstract

**Introduction:**

Senior Instructors and Assistant Professors in their first academic appointment may not have all the tools for an efficient start to their careers. Although many institutions provide access to mentoring programs and seminars on faculty development, the timing and format of the offerings often conflict with ongoing responsibilities of the faculty, particularly clinical faculty.

**Methods:**

We established a collaboration between the Clinical and Translational Science Institute (CTSI) and the University of Rochester Medical Center Office for Faculty Development with the goal of developing a week-long Junior Faculty Core Curriculum that would better suit faculty schedules. We convened focus groups and with their help, identified themes for inclusion in the course. Speakers were identified from among local senior faculty. University leadership was enlisted in promoting the course. Individual speakers and course content were evaluated daily, at the end of the week-long course, and 6 months later. Planning for subsequent years incorporated the feedback. Yearly evaluations and subsequent course modification continued.

**Results:**

Junior faculty from nearly every department in the Medical Center were represented. There was high learner satisfaction and participation however several limitations were identified and addressed in subsequent years. The focus on principles and available resources, not specific skills or content was appropriate. Daily interactions among participants from a wide variety of departments fostered networking among faculty who may not otherwise have met and discussed common interests

**Conclusions:**

The ultimate value of such an early, intensive faculty development program will depend on whether it equips junior faculty to organize, develop, and achieve their academic goals better than alternative formats. This will require further study.

## Introduction

Junior faculty are often successful graduates of high-quality training programs, but such training programs often fail to address the transition to the faculty role and the added expectations that accompany a faculty position. Junior faculty may not know how to proceed efficiently and effectively toward their academic goals, including obtaining extramural funding. To address this need, junior faculty mentorship programs are common at many institutions [1–4]. However, the timing and format of the offerings often conflict with the ongoing responsibilities of the junior faculty, particularly clinical responsibilities. As a result, faculty may not take advantage of what is available.

We set out to provide an alternative approach to a structured core curriculum for junior faculty that would enable them to obtain basic skills and knowledge to enhance their careers and facilitate more rapid progress along their chosen trajectory. To be successful, the curriculum has to include relevant content and be accessible to the target audience. In addition, by bringing together junior and curriculum faculty, collaborations may be established earlier and networking may be facilitated.

In 2012, we established a collaboration between the Clinical and Translational Science Institute (CTSI) and the University of Rochester Medical Center Office for Faculty Development with the goal of developing a Junior Faculty Academic Core Curriculum (JFACC). The costs associated with this curriculum were covered by the CTSI as part of their educational mission. This report describes the steps taken to initiate, implement, and evaluate this program, as well as the ongoing modification process.

## Needs Assessment: Focus Groups

Our first step in developing the curriculum was convening focus groups to help us identify knowledge gaps as well as approaches to presentation of course material. Both junior faculty and experienced faculty mentors were identified who represented 5 departments at the Medical Center and included individuals pursuing basic science, translational, and clinical research and educational scholarship. We felt that it was important to include junior faculty who had been in their roles for 2–3 years and who may have had time to identify deficiencies and difficulties in their transition to academic faculty. More senior faculty, who could reflect on the needs of their current junior associates and on their own careers, were also contacted. We anticipated that their insights on “what I wish I had known earlier in my career” would include topics not considered by junior faculty.

Each focus group consisted of 4–6 individuals with a range of backgrounds and interests. Junior and senior faculty were interviewed separately to enhance communication by junior faculty. We were also able to obtain insights into the strategies that would be most successful for “Generation X,” as these may differ from strategies that worked for the “Baby Boom Generation” [5]. With the assistance of an experienced facilitator, we identified topics that should be included in the curriculum as well as the format of the course. Table 1 includes the outline the facilitator used. A scribe was present to take notes. These notes were later reviewed and formed the basis of a needs assessment report for the course developers.

**Table 1:** Potential questions/themes for discussion

∙ Have you attended any of the Faculty Development Workshops in the past 1–2 years?
◦ If no, why not? (eg, bad timing, topics not of interest)
◦ If yes, were they helpful?
▪ If helpful, which ones, why?
▪ If not helpful, why not?
◦ Other comments about existing workshops
∙ Are there other resources (especially courses, seminar series) you
◦ Would have liked to take advantage of but didn’t?
▪ Which ones?
▪ Why didn’t you attend?
◦ Did take advantage of and found
▪ Valuable—which, why
▪ Not valuable—which, why
∙ In what general spheres do you feel the least prepared with respect to advancing your academic career?
◦ Specific skills (eg, technical skills in the laboratory, statistical analysis/how to talk to a statistician, how to work with the Institutional Review Board [IRB] or University Committee on Animal Resources [UCAR] most efficiently)
◦ Awareness of the promotion requirements and process
▪ For the Researcher/Clinician/Teacher (RCT) track
▪ For the Teacher/Clinician/Scholar (TCS) track
▪ For the Clinician/Teacher (CT) track
◦ Types of grants appropriate for you at this stage in your career—and how to decide which are appropriate and attainable
▪ Local/University of Rochester sponsored
▪ Foundation
▪ Mentored
▪ Independent
◦ How to network—locally and with investigators outside UR
◦ How to “promote” oneself
◦ How to achieve independence—process and timing
◦ How to manage work-life balance
∙ Are you aware of any programs at other institutions that you would like to see developed here?
∙ What other needs do you have and how can we help?

## Curriculum Development

The University of Rochester School of Medicine and Dentistry employs ~1600 individuals whose career trajectories include clinician educators, with primarily clinical and teaching responsibilities; clinician scholars, with clinical responsibilities and participation in clinical or basic science research, but without ongoing, consistent extramural funding, often as part of a collaborative or as a co-investigator; and clinician researchers, with more limited clinical responsibilities and a well-established, consistently funded research program. In addition, Ph.D. faculty are an integral part of the mission of the University, with research and teaching expectations.

Serving this diverse group of individuals was a challenge. For the first year of the curriculum, we made the decision to limit the participants to only those with clinical responsibilities. In the second year, we opened registration to all medical school faculty.

A small committee, consisting of the course director, the Chair of the CTSI Education Directorate, and the Associate Dean for Faculty Development, met and considered the input from the focus groups and the recent educational offerings from the Office for Faculty Development. Other published curricula were also reviewed for common elements.

There were some clear themes and obvious choices for inclusion in the curriculum. An initial draft outline of a week’s worth of sessions was presented to the Education Directorate of the CTSI for input. One charge of this committee is to provide a comprehensive range of didactic programs, including Master’s and Doctoral degree, courses, workshops, and seminar series to serve the educational needs of clinical and translational research professionals at all stages of training and career development. This course addressed part of the Directorate’s mission. After discussion and several drafts, the final topics were identified and prioritized. We based our choices on the assumption that attendees would represent each of the 3 career trajectories for clinicians in approximately equal proportions. Thus, we included sessions on effective teaching, research methods, and funding, as well as life-work balance.

Once the topics were identified, we contacted senior faculty and staff from across the Medical Center to solicit speakers. As some of the topics were similar to those previously presented within one of the faculty development seminar series, we were able to quickly recruit faculty whose presentations had been well-attended and well-received. For other topics, such as “Manuscript Review,” faculty at our institution who were journal editors were an obvious pool of individuals upon which to draw. All faculty enthusiastically gave their time to participate. All participating faculty were asked to provide resource material (slides, references, Web sites) for “students” to use. We utilized DropBox for easy access to this material before, during, and after the course.

To address the issue of conflict with other responsibilities, we designed the curriculum as a full-week course, 9:00 am to 5:00 pm. Nearly all faculty are able to take advantage of out-of-town meetings in their areas of interest and block off time from their schedule to attend these conferences. By structuring this course similarly, and with sufficient lead time, faculty could similarly arrange their clinical responsibilities, and thus “protect” the time necessary to attend the sessions. We chose a week in early fall after new faculty had had a chance to “settle in,” summer vacations were over, and the academic year was beginning.

The first year’s curriculum is outlined in Table 2. A “working lunch” was an integral part of the curriculum to allow networking among participants and course faculty. A 90-minute lunch break allowed for a 30-minute break for participants to catch up on email, phone calls, and other daily obligations to minimize such distractions during the sessions. Junior faculty participants were required to attend all sessions, including working lunches.

**Table 2:** Year 1 Junior Faculty Academic Core Curriculum

Days	Morning session (9:00 am–12:00 pm)	Working lunch (12:00 pm–1:30 pm)	Afternoon session (1:30 pm–5:00 pm)
Monday	Welcome/overview Goals for week Statistics overview Talking to a statistician Formulating a research question Research study designs		Informatics Database development Data management Using eRecord for research
Tuesday	Manuscript review Didactic Anatomy of a paper Peer review process Discussion of reviews—small groups		Career advancement Framing your career Academic portfolio/CV Promotion/tenure Training opportunities Leadership
Wednesday	Grant review Assessing SA Assessing significance Assessing methods		Mock study section Presentation and discussion of 3 different grants (basic science, translational, clinical)
Thursday	“What I wish I had known”: Interviewing/hiring staff/evaluations Locally available resources Budgeting/finance Policies/regulations		“What I wish I had known—continued”
Friday	Mentor/mentee Identifying a mentor Being a protégé Separating Networking Selling oneself Work/life balance		Examples of successful careers in each of the academic tracks Wrap-up Completion of evaluations

## “Selling the Curriculum”

To be successful, the new curriculum had to be accepted and promoted by the Medical Center leadership, principally Department Chairs and Section Chiefs. Department Chairs had to see the value in the course and agree to “protect” the time of their junior faculty to allow them to participate. To this end, the Chair of the Education Directorate, himself a Department Chair, presented the curriculum at a monthly meeting of Department Chairs and Deans of the medical center. The response was overwhelmingly positive as each Chair recognized the importance of a strong academic foundation for junior faculty.

The course was advertised 6 months in advance to potential participants via email announcements, flyers at other faculty development seminars, and by word-of-mouth. Electronic registration was open in early summer, with a deadline ~1 month before the course was to begin. At the time of registration, individuals were asked to identify their anticipated career trajectory. This allowed course directors and speakers to finalize both physical space arrangements and lecture content.

For the first year, we estimated participation based on attendance at previous faculty development seminars of 20–25 persons. However, it quickly became obvious that we were going to exceed this figure and ultimately, because of space constraints, we limited enrollment to 50. The large pool of registrants suggested a large “prevalence” of persons seeking such information, but it remained uncertain what the “incident” pool would be in subsequent years. We asked each enrollee to provide us with the name of their home department and their faculty rank. Nearly every department in the Medical Center was represented—including Medicine, Pediatrics, Surgery, Psychiatry, Neurology, and Dentistry.

## Evaluating the Curriculum

Feedback from participants was important, as we wanted to assure that we were meeting the needs of junior faculty; we therefore sought their input throughout the course. At the end of each day, participants were asked to complete electronic evaluations of each topic and each speaker. At the end of the week, summative evaluations were distributed electronically for additional overall feedback. In addition, we distributed a final evaluation to participants ~6 months after the end of the course to provide an opportunity to reflect on the course content and how they used their new skills gained through participation in the curriculum. Upon completion of the 6-month evaluation, we distributed certificates of completion of the JFACC. Sample items from these evaluations are shown in Table 3. A summary of the evaluations is presented in Table 4.

**Table 3:** Evaluation content

Daily Evaluations
Please indicate if the Learning Objectives were met
Please rate each speaker on a scale of 1 to 5 on
∙ Relevance of their topic
∙ Content of the presentation
∙ Teaching effectiveness
∙ Materials (slides, handouts)
Comment on
∙ Top 3 things learned beyond the objectives
∙ How you will incorporate the information into your career development plan
∙ How we can improve this session
∙ Other comments
Overall Evaluation
1. In what ways did the Academic Core Curriculum for Junior Faculty MEET your expectations?
2. In what ways did the Academic Core Curriculum for Junior Faculty NOT MEET your expectations?
Please comment on areas such as presentations, content, format, venue, course organization, and/or course materials. Which sessions were of the least value and might be eliminated in the future, and what additional sessions would you suggest we include in the future?
3. Other comments and suggestions:
6 mo Overall Evaluation
Please indicate at least 2 ways, over the past 6 mo, in which you put to use information learned through the Junior Faculty Core Curriculum
1.
2.
3.
4.
Upon reflection, are there areas/topics that you wish had been
Included_________________________________________
Excluded_________________________________________

**Table 4:** Summary of overall strengths, limitations, and opportunities based on evaluations of year 1.

Strengths
∙ *Enrollment of 51 junior faculty (60% women and 40% men) in a week-long course (Chair-approved protected time) from 15 Medical Center departments*
∙ *Daily/concurrent evaluations of participants attending JFACC* were uniformly very positive, especially for interactive sessions
∙ *6-mo evaluations (post-JFACC curriculum)* confirmed the value of the curriculum with participants reporting applying the skills and knowledge attained
Participants reported
a) Greater focus on development of their academic career plan
b) Seeking additional mentorship
c) Greater ease in networking
d) Getting “credit” for their educational activities
e) Greater awareness of the range of resources available across the Medical Center
∙ *Perceived value of networking lunches with senior and peer faculty*
Limitations
∙ *Participants limited to only those with clinical responsibilities:* heavily skewed to those planning to pursue either clinical research or educator roles; a few reported planning to pursue basic science research
∙ *Wide spectrum of skills and knowledge:* individual sessions reported as both *too advanced and too basic*
∙ Some sessions likely more appropriate for participants at a somewhat later stage in their career
Opportunities
∙ *Expansion of eligible participants* to include junior faculty without clinical responsibilities (research faculty)
∙ *Networking among clinical and research faculty* may be facilitated
∙ *Expansion to participants from other institutions*
∙ *Potential development of a curriculum designed for later stages of faculty careers*

Although there was high learner satisfaction and participation in its first year, we recognized several limitations. During the development phase, we assumed that participants would represent potential career trajectories in approximately equal numbers. However, participants were heavily skewed to those planning to pursue either clinical research or educator roles; a few clinicians reported plans to pursue basic science research. Even among this relatively homogenous group, there was a wide spectrum of skills and knowledge. Individual sessions were perceived as too advanced by some and too basic by others. There were some topics that most suggested were more applicable for faculty at later stages of their career.

After personally attending each of the sessions, the course director recognized that the majority of sessions were applicable to both faculty with clinical responsibilities and junior faculty in the medical center without clinical responsibilities. The evaluations and observations provided the opportunity to modify the curriculum for the next year and to continue to evaluate its acceptance and success in achieving our goals. The challenge was to improve the alignment of the curriculum focus with participant needs.

## Year 2 Planning and Program Improvement

Planning for the second annual course started right after the initial offering was over. Areas of strength and opportunity for improvement were assessed on the basis of course evaluations, in terms of both subject matter and speakers. The first decision we made was to include Ph.D. (research) junior faculty. The needs of junior faculty, whether or not they had clinical responsibilities, are similar in many ways. Knowledge of the resources available locally and the ability to network effectively are key to academic success, and these were among the cornerstones of our curriculum. We realized, however, that faculty intending to pursue extramural funding of their research endeavors and those planning to concentrate on provision of clinical care and education of the next generation of medical care providers may have different needs. To address this, we chose to provide concurrent sessions one day: one session primarily focused on the grant application process and the other on the clinician educator’s portfolio and educational scholarship (Table 5).

**Table 5:** Year 2 curriculum modifications

∙ WEDNESDAY—CONCURRENT SESSIONS	
TIME	RESEARCHERS
9:00 am	Grant review
	∙ Assessing specific aims
	∙ Assessing significance
	∙ Assessing methods
12:00 pm	WORKING LUNCH
1:30 pm	Research funding
	∙ Sources of funding (federal, industry, foundations)
	∙ Types of grants (K-awards, R01, etc.)
	∙ Reading and interpreting RFAs
	Study Section—what you need to know
TIME	EDUCATORS
9:00 am	Advancing your career as an educator:
	∙ Transforming educational activities into scholarship
	∙ The educator’s portfolio
12:00 pm	WORKING LUNCH
1:30 pm	Advancing your career as an educator—continued
∙ Expansion of sessions deemed of particular value to Year 1 participants	
◦ Time management, resilience/preventing burnout	
◦ University of Rochester, School of Medicine and Dentistry resources (additional speakers; briefer individual presentations)	
∙ Reordering of sessions for better flow	

As we enlisted speakers and facilitators, the majority of whom participated in the first year’s course, we provided specific feedback on content and effectiveness. All of our speakers discussed their evaluations with the course director, and modified their approach as needed. Additional faculty were enlisted to cover areas that were identified by the first year’s participants as “missing” from the curriculum. Year 1 participants were invited to attend the working lunches to share their experiences and application of the knowledge and skills gained through the JFACC course.

In order to provide the appropriate physical space for concurrent sessions, we asked year 2 participants to provide their home department and faculty rank and their intended career trajectory: basic science or clinical research (with the intent to pursue independent extramural funding) versus clinical education. Although the first year’s participants were heavily weighted toward the clinician educator role (~75%–80% of the participants), researchers and clinician researchers predominated in the second year. This was expected, because of the addition of Ph.D. faculty; in addition, a greater proportion of the clinical faculty who participated self-identified as researchers.

At the suggestion of our speakers and facilitators, we also asked the enrollees to let us know what they hoped to get out of each of the sessions. For example, the senior faculty facilitating the session on manuscript review, from the perspective of the journal editor, asked “What is the one question you’d like to ask the editor of the journal to which you submit your manuscripts?” They then used these questions to focus their discussion during the course.

The course was filled well before the deadline for application. We received applications from a wide variety of departments and from both Ph.D. and clinical faculty in approximately equal numbers.

## Year 2 Evaluation

As in year 1, participants were sent daily evaluations in addition to an overall evaluation at the end of the week and a 6-month follow-up evaluation. The responses were again uniformly positive and, in content, similar to year 1. In particular, participants commented that they learned valuable information about resources at the institution for research support and about grant writing and funding sources of which they previously were unaware. They were also grateful for the opportunity to interact and network with their peers and anticipated forming collaborations. Again, there were comments pointing out the wide range of backgrounds and level of knowledge, particularly regarding biostatistics, even though we attempted to address this by dividing the group into 2, one with a statistician who focused on clinical/quality improvement-related research and the other with a statistician who focused on concepts important in the laboratory.

One of the major changes we made was the inclusion of concurrent sessions for those planning to pursue an extramurally funded research career and those anticipating primarily collaborative research, clinical work, and teaching. This change led to an unanticipated dilemma for junior faculty who felt they would benefit from information on both National Institutes of Health (NIH) funding and on developing an educator’s portfolio.

After 6 months, participants were asked to indicate 2 ways that the information learned during the week was used, and whether there were topic areas that should be included or excluded in future sessions. For example, second-year participants were introduced to i2b2 software, an open-source software system that allows users to query clinical data to estimate the size of cohorts available for specific studies. Multiple participants responded that they had utilized the tool to plan their research. The session on mindfulness and work-life balance also resonated with participants: one person had incorporated meditation into the daily schedule, whereas another applied learned skills when making career decisions. Multiple participants indicated that they had developed more detailed career development and mentoring plans. A few participants utilized resources through the CTSI, including voucher support for statistics and an application for CTSI grant funding.

Participants reiterated their desire to participate in sessions on grant funding in addition to sessions on effective teaching and the educator’s portfolio. They requested more time devoted to grant funding, specifically in the areas of grant administration, and requested guidance on NIH funding priorities. They suggested that sharing their own specific aims and getting feedback would have been valuable. Most felt that nothing should be excluded from the course in coming years, although a few suggested condensing the biostatistics and bioinformatics sessions.

## Overall Lessons Learned/Plans for the Future

Our alternative course for junior faculty development was enthusiastically received by participants and by supervising senior faculty. The focus on principles and on available resources, as opposed to specific skills or content, was appropriate: junior faculty often focus on identifying individuals who are experts but are unaware of sources of additional expertise and networking opportunities. An intensive, 5-day course promoted interactions among participants that do not ordinarily occur during intermittent, 1–3 hours faculty development offerings.

We plan to continue to offer this curriculum annually to all junior faculty at the medical school. We will modify the course content and format to respond to the needs of junior faculty from all disciplines. In response to the suggestion that information on grant funding is necessary for the majority of faculty, we will add a session on obtaining funding from societies and foundations. These sources of support are important at the beginning of one’s career to provide the means to collect pilot data for subsequent NIH applications and for those embarking on a more limited research program. Other modifications will reflect new resources available at our institution and the inclusion of speakers who were particularly effective in other faculty development venues. The ultimate value of such an early intensive faculty development program will depend on whether it equips junior faculty to better organize, develop, and achieve their academic goals better than alternative formats. This will require further study. We plan to collect information from participants at 5-year intervals on longevity at the University, promotion success, publications, and grant funding.

Days-long educational offerings may also appeal to mid-career and later-career faculty. We hope to develop a curriculum to meet the needs of Associate Professors as they transition to leadership roles, attempt to expand their funding, try to reinvigorate their careers or develop new initiatives, and develop their mentorship skills. The challenges facing Professors include these areas as well as the transition to retirement and post-retirement opportunities. As was the case with junior faculty, we anticipate that the opportunity to network and learn from each other will be facilitated by a 2–3-day, 9:00 am–5:00 pm course. With the continued support of the CTSI and the Office for Faculty Development, we hope to provide all faculty with access to tools for a more successful, fulfilling career.
